# Polyclonal LC3B Antibodies Generate Non-Specific Staining in the Nucleus of Herpes Simplex Virus Type 1-Infected Cells: Caution in the Interpretation of LC3 Staining in the Immunofluorescence Analysis of Viral Infections

**DOI:** 10.3390/ijms26146682

**Published:** 2025-07-11

**Authors:** Inés Ripa, Sabina Andreu, Daniel Galdo, Oliver Caballero, Raquel Bello-Morales, José Antonio López-Guerrero

**Affiliations:** 1 Department of Molecular Biology, Universidad Autónoma de Madrid, 28049 Madrid, Spain; ines.ripa@cbm.csic.es (I.R.); sandreu@cbm.csic.es (S.A.); daniel.galdo@uam.es (D.G.); oliver.caballero@estudiante.uam.es (O.C.); 2 Centro de Biología Molecular Severo Ochoa (Consejo Superior de Investigaciones Científicas), 28049 Madrid, Spain

**Keywords:** LC3 antibody, herpes simplex virus type 1, autophagy, replication compartment, oligodendrocyte

## Abstract

The most common marker used to monitor autophagy is the microtubule-associated protein light chain 3 (LC3). Upon induction of autophagy, LC3 is conjugated to phosphatidylethanolamine and targeted to autophagic membranes, which can be easily detected by immunofluorescence. However, this technique has some limitations. During the early stages of HSV-1 infection, strong LC3B nuclear staining is observed within the viral replication compartments. This staining is only detected when using polyclonal antibodies. It is noteworthy that monoclonal antibodies or the GFP-LC3 plasmid do not reveal any nuclear LC3 staining. Interestingly, LC3B is not detected in the nuclear fraction of infected cells by Western blotting, even when polyclonal antibodies are used. In infected LC3B knockout cells, nuclear staining is still observed when using polyclonal LC3B antibodies. This suggests that polyclonal LC3B antibodies generate non-specific nuclear staining in infected cells, which could result in misinterpretation and erroneous conclusions. These findings raise questions about the reliability of LC3-immunofluorescence assays in herpesvirus infections. It is imperative that the methodology employed for monitoring autophagy by immunofluorescence in viral infections be reviewed and updated, and that the specificity of anti-LC3B antibodies be tested before use. To ensure the accuracy of the results, it is essential to validate this technique with additional assays, such as by immunoblot analysis or via the use of autophagy-deficient cell lines.

## 1. Introduction

Macroautophagy, hereafter referred to as autophagy, is a conserved and highly regulated process involved in the degradation and recycling of cellular components. The autophagic flux is initiated by the engulfment of cytosolic components in a crescent-shaped structure, the phagophore, that expands and fuses to generate a double-membrane vesicle known as an autophagosome. Autophagosomes are then fused with lysosomes/vacuoles to degrade their content [1].

The formation and maturation of autophagosomes is mediated by the ubiquitin-like LC3 protein family [2,3]. The soluble form, LC3-I, is generated by proteolytic cleavage of pro-LC3 by the ATG4 protease, which exposes a C-terminal glycine that is susceptible to conjugation. ATG7 (E1-like enzyme), ATG3 (E2-like enzyme), and the ATG5-12-16L1 complex (E3-like enzyme) then conjugate LC3-I to phosphatidylethanolamine (PE) on the surface of nascent autophagosomes [4]. The resulting LC3-PE is a membrane-bound form of LC3, known as LC3-II, which is present in both the inner and outer autophagosomal membranes. LC3-II from the inner autophagosomal membrane undergoes acidic lysosomal degradation, whereas ATG4B delipidates LC3-II from the outer membrane to be re-used in the next round of autophagosome formation [5].

Due to the correlation between LC3 and autophagosomal membranes [6], LC3 members are key molecules for monitoring autophagy [7]. One approach for autophagy monitorization is to measure the protein levels of LC3-II by immunoblot analysis. LC3-II has a faster mobility than LC3-I on SDS PAGE, which allows both forms to be differentiated as separate bands [8]. Another approach to measuring autophagosomal membranes is the analysis of LC3B puncta by immunofluorescence or GFP-based microscopy. Furthermore, the autophagic flux can be monitored by the GFP and RFP tandemly tagged LC3 (tfLC3) method [9].

Although LC3 participates in autophagosome biogenesis in the cytoplasm, this protein has also been detected in the nucleus in multiple studies [10,11,12]. Under nutrient-rich conditions, nuclear LC3 associates with high-molecular-weight complexes that localize to the nucleolus [13]. During starvation, deacetylation of nuclear LC3 facilitates its distribution from the nucleus to the cytosol, where it is associated with the autophagy machinery [14,15]. It has been also suggested that the pool of nuclear LC3 plays different roles in the nucleus of cells under certain stresses. For instance, LC3 can facilitate the degradation of the nuclear lamina in response to oncogenic induction [16]. Indeed, LC3 is translocated to the nucleolus in association with a selective receptor of ribophagy in response to cyclic mechanical stress [17] and has been found to interact with phosphorylated ERK in the nucleus after epidermal growth factor (EGF) exposure [18]. It has been suggested that de novo LC3 lipidation can occur in the nucleus, and the presence of autophagy proteins required for PE conjugation, such as ATG7, ATG5–ATG12, ATG16, as well as ATG4B, which recycles LC3-I, and the autophagy inducer ULK1 have been found in nuclear fractions from mouse livers [18].

The human LC3 subfamily comprises three homologous isoforms—LC3A, LC3B, and LC3C—with LC3B being the most common and best understood autophagy marker [10]. Nuclear LC3B puncta have been observed in herpesvirus infections [19,20]. Our group is focused on the study of herpes simplex virus type 1 (HSV-1), a neurotropic virus that, after a primary infection of epithelial cells, traffics from the axon terminal to the trigeminal ganglia [21], where it establishes lifelong latency [22]. To combat autophagy, HSV-1 expresses various anti-autophagic proteins, with the ICP34.5 protein being the first to be characterized [23,24]. ICP34.5 plays a crucial role in the recruitment and sequestration of Beclin-1, preventing the formation of autophagosomes [25]. It is important to note that ICP34.5 is not the only HSV-1 protein capable of inhibiting autophagy, nor is Beclin-1 the only target used by the virus for pathway suppression. Another HSV-1 protein that plays an anti-autophagic role is Us11, which physically interacts with PKR [26], preventing autophagy induction [27,28]. It has been observed that the HSV-1 kinase Us3 may also play a role in inhibiting autophagy [29]. Us3 has been shown to phosphorylate and activate mTORC1 [30], which serves as a negative regulator that keeps autophagy inactive under normal physiological conditions. Furthermore, Us3 has been shown to modulate autophagy through the phosphorylation and inactivation of both the ULK1 complex and Beclin-1 [29].

HSV-1 can occasionally spread to the central nervous system (CNS), where it has been linked to neurodegenerative and demyelinating diseases, such as multiple sclerosis (MS) [31]. In MS lesions, there is a severe reduction in oligodendrocytes (OLs), the myelinating cells of the CNS; this decrease is caused by the failure of the oligodendrocyte precursor cells (OPCs) to proliferate and differentiate [32]. In this work, we observed that polyclonal LC3B antibodies generate strong staining in the nucleus of infected oligodendroglial cell lines and murine cultures upon examination by immunofluorescence analysis. Our previous findings indicate that HSV-1 inhibits autophagy in these cells [33]. Consequently, the strong LC3B signal observed by immunofluorescence was not consistent with the previous results. Validation of those results by Western blotting and LC3B-deficient cells indicated that the nuclear staining observed by immunofluorescence was non-specific. This non-specific staining was dependent on HSV-1 replication, indicating that polyclonal LC3B antibodies are suitable for the immunofluorescence analysis of uninfected cells [34].

Additionally, endogenous LC3 can be incorporated into intracellular protein aggregates, such as inclusion bodies [35], which can be mistaken for autophagosomes. It is important to note that inhibition of autophagy in the later stages of the pathway can also lead to the accumulation of autophagosomes. This accumulation can be misinterpreted if autophagy is only monitored by LC3 immunofluorescence without performing comparative assays with and without the addition of autophagic inhibitors, such as bafilomycin [7]. These observations raise concerns about the reliability of LC3-immunofluorescence assays in HSV-1-infected cells and emphasize the need for validation of the LC3-immunofluorescence results.

## 2. Results

### 2.1. LC3B Staining Is Observed in the Replication Compartments of HSV-1-Infected HOG Cells by Immunofluorescence

**Figure 1:**
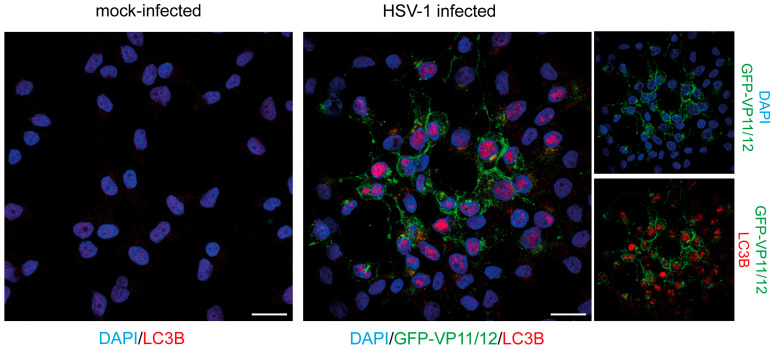
LC3B staining detected by immunofluorescence in the nucleus of HSV-1-infected cells. HOG cells were infected with GHSV-UL46 at an m.o.i of 1. At 24 hpi, cells were fixed and stained using an anti-LC3B antibody (2220SS). Mock-infected cells were used as a control. The immunofluorescence images depict a lysis plaque of HSV-1. A strong nuclear signal was observed in HSV-1-infected cells by using the anti-LC3B polyclonal antibody. No signal was observed in the nucleus of non-infected HOG cells. Scale bar, 20 µm.

To analyze the cellular location of LC3B by immunofluorescence, the human oligodendroglioma-derived cell line HOG was infected with a recombinant HSV-1, known as GHSV-UL46, which expresses the GFP fused to the tegument protein VP11/12. All samples were stained with a polyclonal LC3B antibody. As expected, infected cells did not show a robust LC3B signal in the cytoplasm, since HSV-1 inhibits the formation of autophagosomes [25,36]. However, strong staining was observed in the nucleus of infected cells (Figure 1). During the replication of HSV-1, host chromatin is displaced toward the nuclear envelope, and the center of the nucleus becomes almost entirely devoid of chromatin [37]. The staining generated by the LC3B antibody was particularly pronounced in nuclear regions that were devoid of chromatin and where the DNA-specific DAPI probe failed to stain (Figure 1).

**Figure 2:**
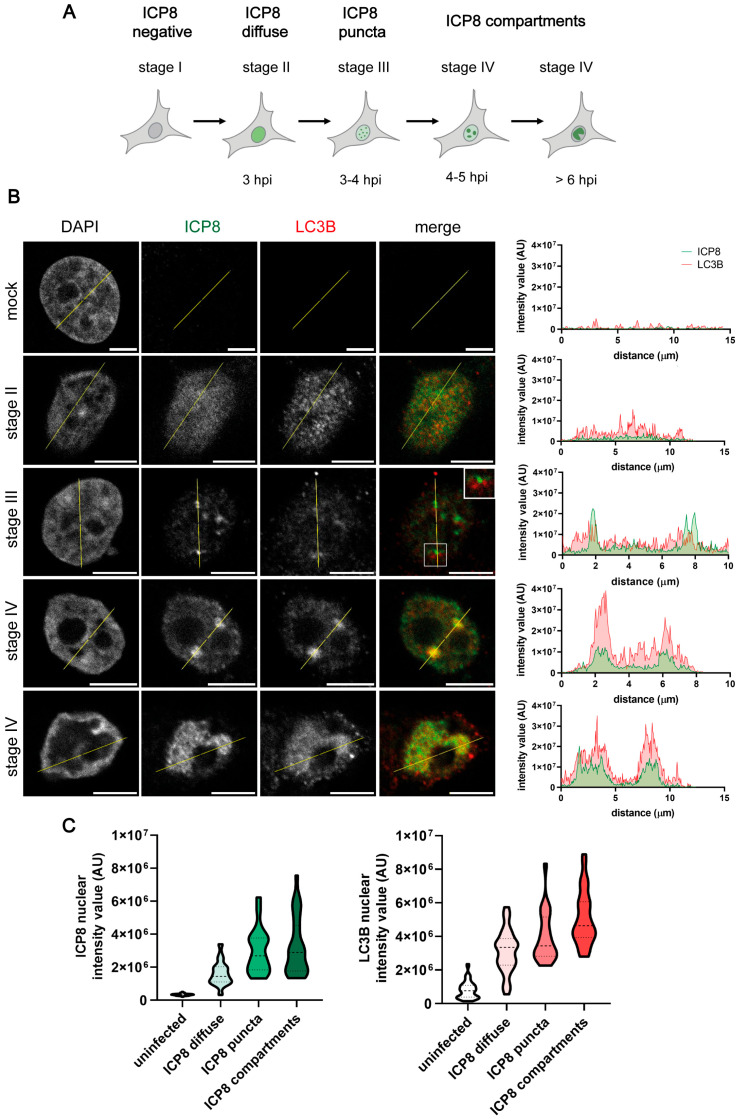
LC3B staining was detected in the RCs of HSV-1-infected HOG cells by immunofluorescence analysis. (**A**) Nuclear distribution of ICP8 during the formation of HSV-1 RCs. The hour post-infection (hpi) at which each stage is formed is indicated [33]. (**B**) Cells were infected with HSV-1 and fixed at 3, 4, and 6 hpi (m.o.i. = 40). All samples were probed with anti-ICP8 and polyclonal anti-LC3B (2220SS) antibodies. Nuclei were labeled with DAPI. The graphs show the intensity values of ICP8 and LC3B fluorescence across the yellow line of the images. AU, arbitrary unit. A concordant profile of the fluorescent signal from ICP8 and LC3B was observed in HSV-1-infected HOG cells. No signal was observed in the nucleus of uninfected cells. Scale bar, 5 µm. (**C**) Intensity values of ICP8 and LC3B fluorescence in the nucleus of mock-infected and infected cells. The intensity of fluorescence was classified into three categories based on the ICP8 signal: diffuse (stage II), puncta (stages III-IV), or compartment (stage IV). To quantify the intensity of fluorescence in the nucleus, the following computer code was used (see Supplementary Information). At least 150 HOG cells from each condition were evaluated. The increase in the intensity of LC3B fluorescence coincided with that of ICP8 during the formation of RCs.

Replication compartments (RCs) of HSV-1 are formed in the nuclear regions devoid of chromatin. RCs are membraneless inclusions within the nucleus that constitute spherical domains where viral gene transcription, DNA replication, and viral genome packaging take place [38,39,40]. Since LC3B has been described as an RNA-binding protein [41], we decided to monitor those viral structures. Using immunofluorescence microscopy, we monitored the formation of RCs by analyzing the nuclear distribution of the major DNA-binding protein ICP8 (Figure 2A) [33] ICP8 staining becomes detectable in stage II of RC formation. In this stage, ICP8 microfoci are formed and this protein can be observed in a diffuse pattern in the nucleus. Cells in stage III display foci of ICP8, which correspond to pre-replicative sites. The coalescence of these small pre-replicative sites leads to the formation of larger punctate foci and, finally, to the generation of large globular structures corresponding to mature RCs (stage IV) (Figure 2A) [42].

While the mock-infected cells displayed no LC3B signal, we observed a diffuse signal for this protein in the nucleus of infected cells during stage II of RC formation. Throughout stage III, LC3B foci were found adjacent to ICP8 foci, which correspond to pre-replicative sites. The intensity of the LC3B signal became particularly high in RCs during stage IV (Figure 2B). These results suggest that LC3B could be recruited in HSV-1 replication structures in HOG cells. By measuring the intensity value of fluorescence within the nucleus of mock-infected and HSV-1-infected cells, we noticed that the intensity of LC3B fluorescence increased concomitantly with that of ICP8 during the formation of RCs (Figure 2C).

LC3 staining can be affected by the conditions of permeabilization. A high percentage of Triton X-100 may cause the formation of LC3 aggregates [43] or promote LC3 nuclear staining [44]. We confirmed that LC3B staining in the nucleus of infected cells was still detectable when lower percentages of Triton X-100 were used (Figure S1).

### 2.2. LC3B Staining Is Detected by Immunofluorescence in the RCs of HSV-1-Infected OPCs

**Figure 3:**
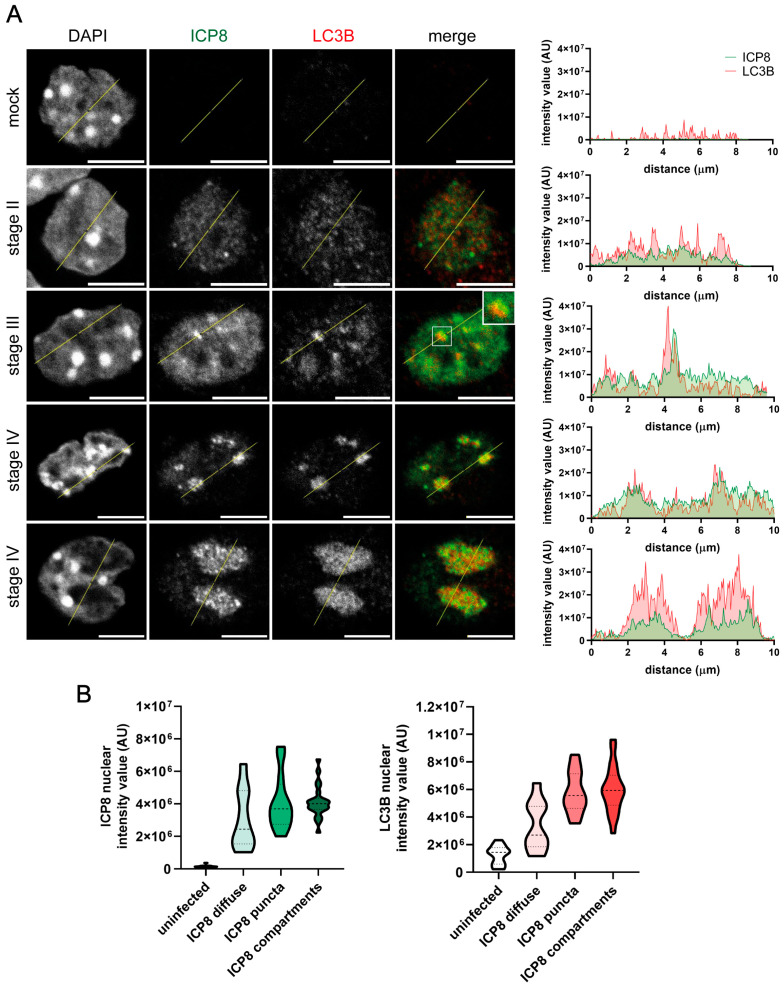
LC3B staining was detected in the HSV-1 RCs of infected OPCs. OPCs were infected with HSV-1 and fixed at 3, 4, and 6 hpi (m.o.i. = 40). All samples were probed with anti-ICP8 and polyclonal anti-LC3B (2220SS) antibodies. Nuclei were labeled with DAPI. (**A**) Immunofluorescence images of the four stages of HSV-1 RC formation. The graphs show the intensity values of ICP8 and LC3B fluorescence across the yellow line of the images. AU, arbitrary unit. A concordant profile of the fluorescent signal from ICP8 and CL3B was observed in HSV-1-infected OPCs. No signal was observed in the nucleus of uninfected OPCs. Scale bar, 5 µm. (**B**) The intensity value of ICP8 and LC3B fluorescence in the nucleus of mock-infected and infected cells was quantified and categorized, based on the ICP8 signal, as diffuse (stage II), puncta (stages III–IV), or compartment (stage IV). To quantify the intensity of fluorescence in the nucleus, the following computer code was used (see Supplementary Information). At least 150 OPCs from each condition were evaluated. The increase in the intensity of LC3B fluorescence coincided with that of ICP8 during the formation of RCs.

Oligodendrocyte precursor cells (OPCs) were isolated from mice and infected with HSV-1. Infected OPCs showed the same LC3B nuclear staining as HOG cells. LC3B was not detectable in the nucleus of mock-infected OPCs, while the intensity of LC3B fluorescence in the nucleus of infected cells was enhanced. LC3B was apparently recruited to the limits of replicative sites during stage III and to the mature RCs that characterize stage IV (Figure 3A). As we observed in the HOG cell line, in infected OPCs, the nuclear intensity of LC3B fluorescence increased concomitantly with that of ICP8 (Figure 3B).

### 2.3. Nuclear LC3B Staining During HSV-1 Infection Is Not Restricted to Oligodendroglial Cells

**Figure 4:**
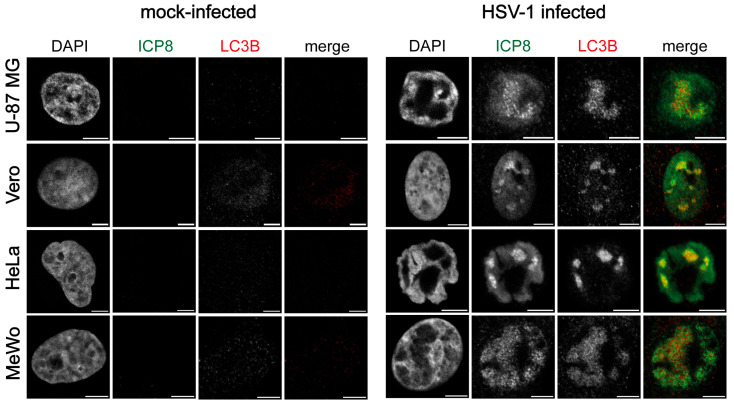
LC3B staining was observed in the HSV-1 RCs of different cell types. Immunofluorescence images of Vero, U-87 MG, HeLa, and MeWo cell lines infected with HSV-1 (m.o.i. = 40) and fixed at 6 hpi. Mock-infected cells were used as a control. All samples were probed with anti-ICP8 and polyclonal anti-LC3B (2220SS; Sigma) antibodies. Nuclei were labeled with DAPI. A strong nuclear signal was observed in HSV-1-infected cells by using the anti-LC3B polyclonal antibody. No signal was observed in non-infected cells. Scale bar, 5 µm.

To assess whether the nuclear detection of LC3B in infected HOG cells and OPCs is specific to OLs, we analyzed LC3B staining during infection in other cell types. For this purpose, we used the U-87 MG cell line, derived from a human astrocytoma; the Vero cell line, from kidney tissue of an African green monkey; the HeLa cell line, from a human adenocarcinoma; and the MeWo cell line, from a human melanoma. Immunofluorescence images showed LC3B staining in the HSV-1 RCs at stage IV, as in our glial cell models, when using a polyclonal anti-LC3b antibody. As seen in HOG cells and OPCs, no LC3B signal was detected in the nucleus of non-infected cells (Figure 4).

### 2.4. Nuclear LC3B Staining of HSV-1-Infected Cells Is Only Detected with Polyclonal Antibodies

**Figure 5:**
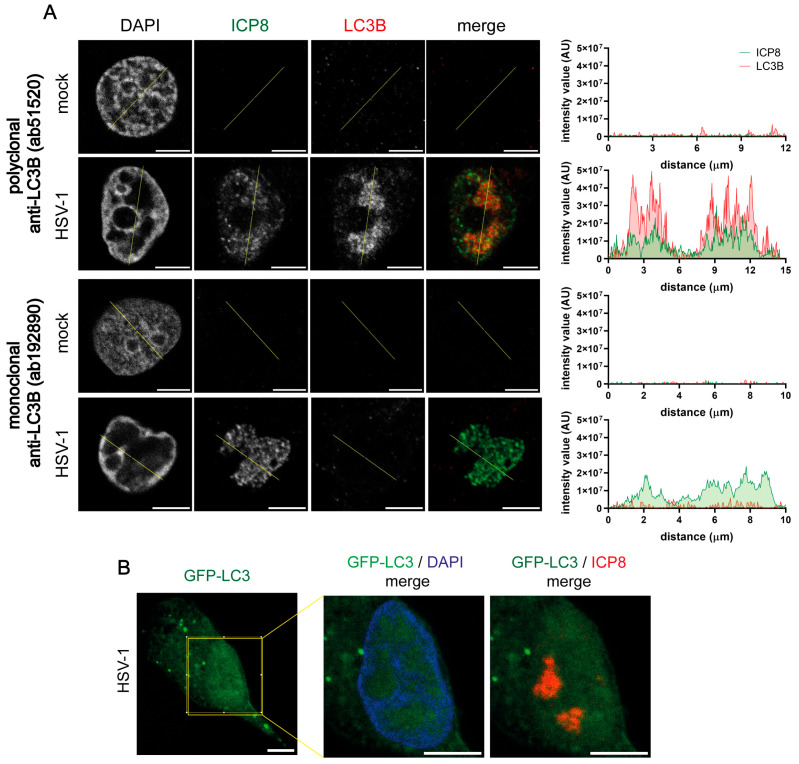
Nuclear LC3B staining in HSV-1-infected cells was only detected with polyclonal LC3B antibodies. (**A**) HOG cells were infected with HSV-1 (m.o.i. = 40) and fixed at 6 hpi. Samples were probed with an anti-ICP8 antibody and with a polyclonal (ab51520) or monoclonal (ab192890) LC3B antibody. The graphs show the intensity values of ICP8 and LC3B fluorescence across the yellow line of the images. AU, arbitrary unit. Non-infected cells did not display any LC3B signal. HSV-1-infected HOG cells revealed an LC3B nuclear signal when utilizing the polyclonal antibody but not the monoclonal one. (**B**) HOG cells were transiently transfected with a GFP-LC3 plasmid. After transfection, the cells were infected with HSV-1 (m.o.i. = 40) and fixed at 6 hpi. RCs of HSV-1 were visualized with anti-ICP8. Nuclei were labeled with DAPI. The presence of GFP-LC3 puncta was observed in the cytoplasm of HSV-1-infected HOG cells, indicating the presence of autolysosomes. However, no GFP-LC3 signal was observed in the nucleus of infected cells. Scale bar, 5 µm.

Some authors reported that different LC3 antibodies led to distinct levels of LC3 nuclear staining, with nuclear LC3 being more easily seen with polyclonal antibodies compared with monoclonal ones [44]. A rabbit polyclonal LC3B (2220SS) antibody was used in the previous experiments. We have repeated these assays in HOG cells using another rabbit polyclonal LC3B antibody (ab51520) and a monoclonal one (ab192890). Nuclear LC3B staining was detected in HSV-1-infected cells with the polyclonal antibody ab192890, whereas no signal was observed using the monoclonal antibody (Figure 5A). These results indicate that the nuclear signal in infected cells was not exclusively detected with the polyclonal LC3B 2220SS antibody. In fact, it was also generated with other polyclonal antibodies against LC3B. This suggests that the generation of the nuclear LC3B signal in infected cells is characteristic of polyclonal antibodies.

Then, we assessed whether nuclear LC3B staining was detectable in infected cells that were transfected with a GFP-LC3B plasmid. We observed the formation of GFP-LC3B puncta in the cytoplasm, which corresponded with autophagosomes. However, we did not observe the presence of GFP-LC3B puncta in the nucleus of infected cells. This result indicates the absence of autophagosomal structures in the nucleus (Figure 5B).

### 2.5. The Isoforms LC3A and LC3C Are Not Detected in the Nucleus of Infected HOG Cells and OPCs

**Figure 6:**
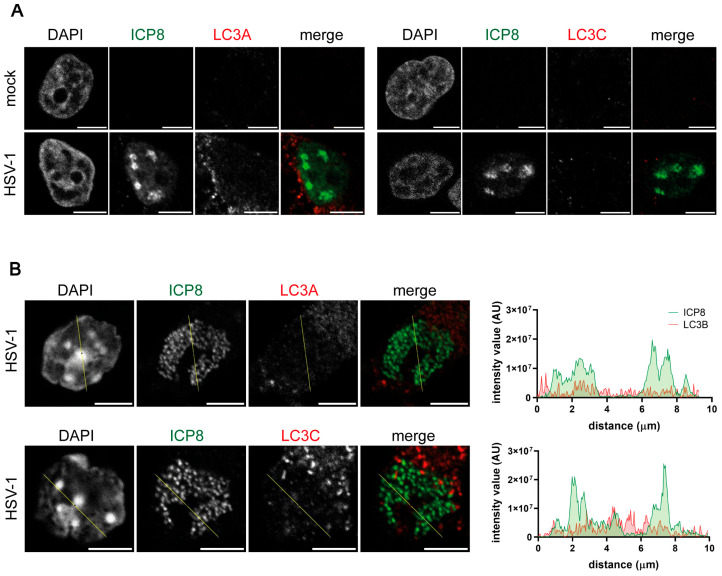
Polyclonal LC3A and LC3C antibodies did not generate a fluorescence signal in the nucleus of HSV-1 infected HOG cells and OPCs. HOG cells (**A**) and OPCs (**B**) were infected with HSV-1 for 6 hpi (m.o.i. = 40). The samples were probed with anti-ICP8 and a polyclonal anti-LC3A (PA5-22990) or anti-LC3C (SAB4200822) antibody. Nuclei were labeled with DAPI. The graphs show the intensity values of ICP8 and LC3B fluorescence across the yellow line of the images. AU, arbitrary unit. No LC3A or LC3C fluorescence signal was detected in the nucleus of infected and non-infected cells. Scale bar, 5 µm.

The LC3 subfamily has three different isoforms: LC3A, LC3B, and LC3C. All the isoforms show a conserved region that is required for autophagosome formation [2], but they have different expression patterns [6] and cellular localization [10,11]. Unlike for LC3B, no signal from either LC3A or LC3C was detected in the nucleus of HSV-1-infected cells using polyclonal antibodies (Figure 6A). Similar results were obtained with the primary cultures of OPCs (Figure 6B).

### 2.6. LC3B Is Not Detected in the Nuclear Fraction of HSV-1-Infected Cells by Immunoblotting

**Figure 7:**
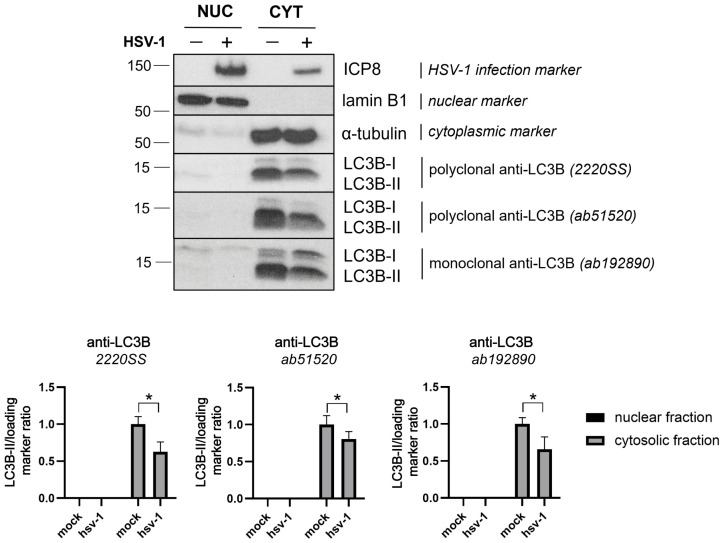
LC3B was not detected in the nuclear fraction of HSV-1 infected cells by immunoblot analysis. HOG cells were infected with HSV-1 (m.o.i. = 40) for 6 hpi. The protein expression levels of LC3B in nuclear and cytosolic fractions of the infected HOG cells were measured by immunoblotting using the polyclonal LC3B antibodies 2220SS and ab51520 and the monoclonal LC3B antibody ab192890. The antibody against ICP8 was used to detect HSV-1 infection. Representative Western blots are shown for LC3B-I/II. The ratios of LC3B-II/lamin B1 and LC3B-II/α-tubulin were calculated for the nuclear and cytosolic fractions, respectively. The data were further normalized to the value of mock-infected cells (set to 1) and reported as the mean ± standard error (SEMs) (*n* = 4). No LC3B protein was detected in the nuclear fraction of HSV-1-infected and mock-infected HOG cells. The LC3B protein was found to be present in the cytoplasm of infected and uninfected cells. The LC3B-II/I ratio was significantly lower during infection, indicating an inhibition of autophagic flux. Significance was evaluated using a two-tailed Mann–Whitney test, which compared each column with its respective mock control. *, *p* < 0.05.

Both LC3-I and LC3-II are generally indistinguishable by immunofluorescence analysis. To determine whether the nuclear LC3B staining corresponds to form I or II, we used ATG5 knockout (KO) cells, which are unable to lipidate LC3B-I. In HSV-1-infected ATG5 KO cells, LC3B staining still appeared in the nucleus, indicating that the nuclear signal did not correlate with LC3B-II and, therefore, did not represent autophagosomes (Figure S2). We then examined LC3B expression levels in the nuclear and cytoplasmic fractions of HSV-1-infected cells by immunoblot analysis. We used lamin B1 and α-tubulin as nuclear and cytoplasmic markers, respectively. They served as suitable loading markers as their expression levels remained unchanged between mock and infected cells (Figure S3). LC3B levels in the cytosolic fractions were lower in infected cells than in the mock control, suggesting a lower accumulation of autophagosomal membranes during the infection. Importantly, LC3B was not detected in the nuclear fraction of infected cells, contradicting the results obtained by immunofluorescence (Figure 7).

### 2.7. The Nuclear Staining Observed with Polyclonal LC3B Antibodies in HSV-1-Infected Cells Appears to Be Non-Specific

**Figure 8:**
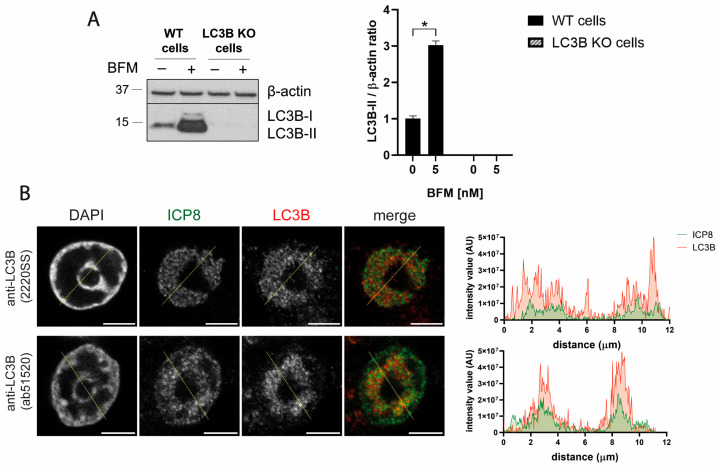
Polyclonal LC3B antibodies generated nuclear staining in HSV-1-infected LC3B KO cells, indicating that this fluorescence signal is non-specific. (**A**) The *LC3B* gene was knocked out in the HOG cell line by transfection of the Cas9:gRNA ribonucleoprotein complex. WT and KO cells were mock-treated and treated with 5 nM BFM (B1793, Sigma) for 24 h, and the absence of LC3B in the KO cells was verified by immunoblot analysis. The data were normalized to the value of mock-treated WT cells (set to 1) and reported as the mean ± SEM. The absence of the LC3B protein in KO HOG cells confirmed the efficacy of the knockout process. Significance was assessed by a two-tailed Mann–Whitney test (*n* = 4). *, *p* < 0.05. (**B**) LC3B KO HOG cells were infected with HSV-1 (m.o.i. = 40) for 6 hpi. The samples were probed with anti-ICP8 and the polyclonal LC3B antibodies 2220SS and ab51520. Nuclei were labeled with DAPI. The graphs show the intensity values of ICP8 and LC3B fluorescence across the yellow line of the images. Nuclear LC3B staining was observed in HSV-1-infected LC3B KO cells, indicating that this fluorescence signal is non-specific. AU, arbitrary unit. Scale bar, 5 µm.

To verify the specificity of the polyclonal LC3B antibodies, we generated LC3B KO HOG cells using the CRISPR/Cas9 system. The absence of LC3B was confirmed by immunoblotting. LC3B was not detected in LC3B KO cells, even after treatment with the inhibitor of lysosomal acidification, bafilomycin A1 (BFM) (Figure 8A). Since LC3B KO cells are deficient in autophagy, a crucial process for homeostasis maintenance and cellular survival, we examined the morphology and the cell viability of these cells. The cell viability of KO cells remained above 90%, and no significant changes were observed in the cellular morphology (Figure S4).

After validating the viability and functionality of these LC3B KO cells, the cells were infected with HSV-1. Immunofluorescence using polyclonal LC3B antibodies revealed an intense staining within the RCs (Figure 8B). These results strongly suggest that the signal generated by polyclonal LC3B antibodies in the nucleus of HSV-1 infected cells is not specific to the LC3B protein.

### 2.8. The Nuclear Staining Detected with Polyclonal LC3B Antibodies in HSV-1-Infected Cells Is Dependent on Viral Replication

**Figure 9:**
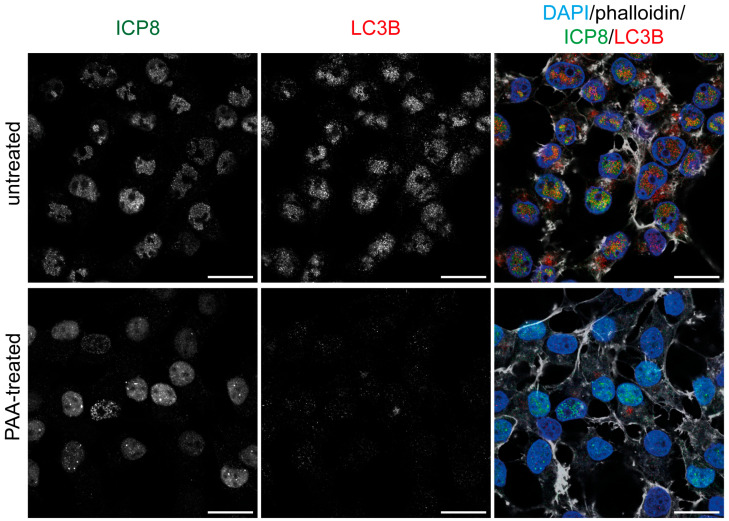
The fluorescence staining detected in the nucleus of infected cells using polyclonal LC3B antibodies is dependent on HSV-1 genome replication. HOG cells were HSV-1 infected (m.o.i. = 40) for 6 hpi. PAA (600 µg/mL) was added to the medium at the time of virus adsorption and maintained throughout the course of the infection. All samples were probed with anti-ICP8 and a polyclonal anti-LC3B antibody (2220SS). Nuclei were labeled with DAPI, and the contour of the cells was visualized with Alexa Fluor 647–phalloidin. No LC3B fluorescence signal was observed in PAA-treated cells, in which viral replication has been inhibited, indicating that the non-specific LC3B nuclear signal observed in untreated infected cells is dependent on HSV-1 genome replication. Scale bar, 20 µm.

To determine whether the LC3B staining was dependent on viral replication, we blocked the replication of the HSV-1 genome by the addition of the viral polymerase inhibitor phosphonoacetic acid (PAA). The inhibition of viral DNA synthesis by PAA prevents the formation of RCs and the marginalization of chromatin to the nuclear periphery [45,46]. Then, HOG cells were mock-treated or PAA-treated, and the LC3B staining was analyzed by immunofluorescence. We confirmed that polyclonal LC3B antibodies did not generate any nuclear staining in PAA-treated cells (Figure S5). Finally, cells were infected with HSV-1 for 6 h in the presence or absence of PAA. Mock-treated cells exhibited the formation of RCs (stage IV) following HSV-1 infection, whereas in cells treated with PAA, RC formation was blocked in stage III, in which HSV-1 prereplicative sites begin to form. No nuclear fluorescence signal was detected with polyclonal LC3B antibodies in PAA-treated infected cells, suggesting its dependence on HSV-1 replication (Figure 9).

### 2.9. Polyclonal LC3B Antibodies Are Appropriate for the Immunofluorescence Analysis of Autophagy in Non-Infected Cells

**Figure 10:**
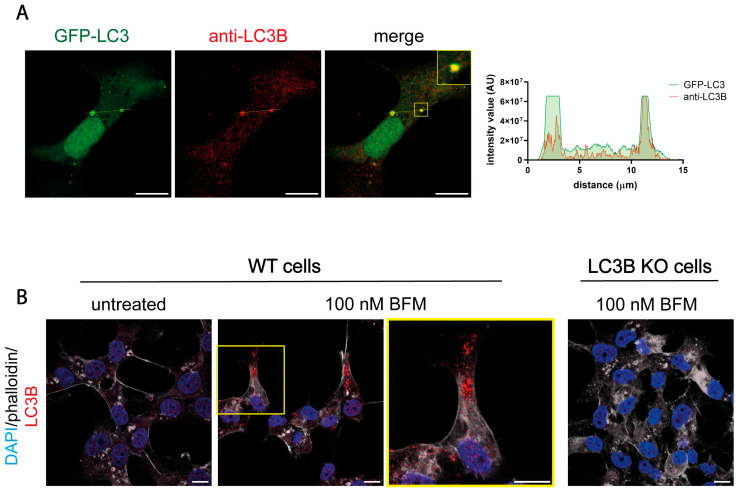
Polyclonal LC3B antibodies are suitable for LC3-immunofluorescence analysis in uninfected cells. (**A**) HOG cells were transiently transfected with a GFP-LC3 plasmid. After transfection, cells were fixed and stained with the polyclonal anti-LC3B antibody (2220SS). The graphs show the intensity value for the GFP-LC3 and endogenous LC3B fluorescence across the yellow line of the images. AU, arbitrary unit. The intensity values for the fluorescence corresponding to GFP-LC3 and endogenous LC3B were found to be concordant, suggesting that the use of polyclonal anti-LC3B was suitable in uninfected cells. (**B**) Cells were mock-treated or treated with 100 nM BFM (B1793; Sigma) for 24 h, and after fixation, they were probed with the anti-LC3B antibody (2220SS). Alexa Fluor 647–phalloidin was used to visualize the contour of the cells, and the nuclei were labeled with DAPI. The addition of bafilomycin led to an increase in LC3B punctate, which resulted from the accumulation of autolysosomes. These results further support the suitability of using polyclonal anti-LC3B in uninfected cells. Scale bar, 10 µm.

Not all antibodies against LC3 are appropriate for immunofluorescence analysis. We assessed whether the polyclonal LC3B antibody (22200S) used in this study was suitable for detecting LC3B by immunofluorescence in uninfected cells. To determine this, we tested the antibody in cells which had been transfected with a GFP-LC3 plasmid. The staining produced by the antibody colocalized with the GFP-LC3 puncta, demonstrating its reliability in detecting LC3B by immunofluorescence analysis (Figure 10A). We then validated the suitability of the antibody to detect autophagosomes in BFM-treated cells. As HOG cells have a low level of basal autophagy, no autophagosomes were detected by immunofluorescence analysis. The accumulation of autophagosomes in the cytosol must be induced by BFM treatment to visualize the autophagosomal membranes (Figure 10B). Autophagosomal membranes were detected in BFM-treated cells using the anti-LC3B polyclonal antibody (Figure 10B), suggesting that it is effective in detecting LC3B and in monitoring autophagy by immunofluorescence in uninfected cells. The antibody did not generate any staining in BFM-treated LC3B KO cells (Figure 10B), which confirms the absence of LC3B in mutant cells and validates the specificity of the staining in BFM-treated WT cells. Polyclonal (ab51520) and monoclonal (ab192890) LC3B antibodies were used to repeat these experiments, and the same results were obtained (Figure S6).

## 3. Discussion

Detection of LC3 by immunofluorescence is one of the most common assays for measuring autophagy. This technique is based on the principle that LC3-II is associated with autophagosomal membranes and appears as distinct puncta in the cell that can be easily quantified. However, an increasing number of authors are urging caution in the interpretation of the results obtained with this technique. First, it is important to confirm the specificity of the antibody used for LC3 staining. It has been reported that some rabbit polyclonal LC3B antibodies show cross-reactivity with the LC3A protein [34]. The specificity of the polyclonal LC3B antibody used in this study (2220SS) had been already verified [11]. Cell permeabilization conditions can also alter the LC3 staining. GFP-LC3 can aggregate into autophagosome-like structures when cells are permeabilized with detergents such as saponin or Triton X-100 [43]. Therefore, puncta containing LC3 do not always represent autophagic structures. Overexpression of GFP-LC3 can also lead to the formation of aggregates in an autophagy-independent manner. Additionally, both GFP-LC3 and endogenous LC3 can be incorporated into intracellular protein aggregates, such as inclusion bodies [35] or aggresome-like induced structures (ALISs) [47]. The reliability of immunofluorescence analysis for autophagy monitorization has been also questioned because in some cases, an increase of LC3-positive puncta is observed when autophagosome formation is impaired. In the absence of LC3-II, the endogenous LC3-I can form puncta with the long-lived protein p62/SQSTM1, which is accumulated when autophagy is impaired [48]. Therefore, LC3-immunofluorescence assays should be carefully interpreted.

In this study, we analyze the reliability of the LC3 nuclear staining observed in HSV-1 infected cells. The use of polyclonal antibodies against the LC3B isoform produced strong staining in the RCs of infected cells. This nuclear staining was not observed using polyclonal antibodies against the LC3A and LC3C isoforms. Previous studies have observed nuclear LC3B staining in varicella–zoster virus (VZV)-infected cells by analyzing both endogenous LC3B [19] and GFP-LC3 puncta [20]. While LC3B antibodies caused prominent LC3 nuclear staining in VZV-infected cells, no nuclear signal was observed with monoclonal antibodies [44]. We obtained similar results when studying endogenous LC3, as nuclear LC3B staining of HSV-1 infected cells was detected with polyclonal antibodies but not with monoclonal ones. We also did not observe GFP-LC3 puncta in the RCs of infected cells.

The authors who investigated the formation of LC3 nuclear staining in VZV-infected cells argued that the nuclear puncta did not represent autophagosomes, as no autophagic structures were found by electron microscopy (EM) [44]. We analyzed the nuclear and cytosolic fractions of HSV-1-infected cells by immunoblotting. However, LC3B was not detected in the nuclear fraction, contradicting the results obtained by immunofluorescence. LC3B KO cells were generated using the CRISPR/Cas9 system, and nuclear LC3B staining was analyzed by immunofluorescence. Similarly to WT cells, strong staining of the RCs was still detectable when polyclonal LC3B antibodies were used. These results suggest that the nuclear staining observed with polyclonal LC3B antibodies in HSV-1 infected cells was non-specific. When HSV-1 replication was blocked with PAA, nuclear LC3B staining was not observed. These results indicate that the non-specific signal generated by LC3B antibodies is dependent on HSV-1 genome replication. In the absence of infection, these antibodies specifically recognize LC3B, making them appropriate for the detection of autophagosomes.

Further research is needed to determine the reason for the lack of specificity of LC3B polyclonal antibodies in immunofluorescence assays of HSV-1-infected cells. The polyclonal antibodies may be cross-linking with viral antigens and/or with cellular proteins that are highly abundant in RCs. During the transcription and replication of HSV-1, protein aggregates are observed in the cell nucleus. These aggregates include viral transcription and replication factors, such as ICP8, ICP4, and viral DNA polymerase protein, as well as cellular proteins involved in DNA processing and the DNA-damage response [49]. These aggregates could be interacting with the LC3B antibody, “trapping” it. This would not be feasible with techniques such as Western blotting, in which proteins are denatured.

The polyclonal antibody against LC3B (2220SS), which was primarily used during the experiments, has been previously validated [11] and has been demonstrated to not be cross-reactive with the LC3A isoform. This isoform shows the most significant similarity to LC3B. Therefore, if the antibody did not recognize LC3A, it is unlikely that it would recognize other proteins due to its similar homology [2]. Furthermore, the generation of LC3-punctate fluorescence that does not represent autophagosomes had already been observed in herpes zoster infection [44]. These findings indicate that the generation of non-specific fluorescence signals by LC3 polyclonal antibodies may also occur in other herpesvirus infections and even in those of other viral species.

In summary, it is advisable to take caution when interpreting LC3-immunofluorescence in herpesvirus-infected cells. The use of polyclonal LC3B antibodies to monitor autophagy by immunofluorescence in HSV-1-infected cells has been shown to lead to a non-specific nuclear signal. This could potentially result in misinterpretation and erroneous conclusions. This non-specific signal is not generated by the use of a GFP-LC3 plasmid or LC3B monoclonal antibodies. However, while the transfection of the GFP-LC3 plasmid is highly effective for the quantification of autophagosomes by confocal microscopy in herpes infections, staining with the LC3B monoclonal antibody did not produce any fluorescent signal, whether nuclear or cytoplasmatic. Therefore, overexpressing LC3B using the LC3-GFP plasmid is necessary to detect the presence of autophagosomes in HSV-1 infections by confocal microscopy. In addition, it is advisable to verify the results of immunofluorescence using other methods, such as by analyzing LC3-II using a Western blot or by generating autophagy-deficient cells.

## 4. Materials and Methods

### 4.1. Cell Lines

The HOG cell line was provided by A. T. Campagnoni (University of California, Los Angeles, CA, USA). The Vero cell line was provided by Enrique Tabarés (Universidad Autónoma de Madrid, Spain). The MeWo cell line was provided by L. Montoliu (CNB, CSIC, Madrid, Spain). The HeLa cell line was a gift of J. M. Almendral (CBMSO, CSIC, Madrid, Spain). These cell lines were cultured in low-glucose Dulbecco’s Modified Eagle’s Medium (DMEM) containing 10% (vol/vol) fetal bovine serum (FBS), 2 mM glutamine, penicillin (50 U/mL), and streptomycin (50 µg/mL). The U-87 MG cell line, kindly provided by María Jesús Bullido (CBMSO, Madrid, Spain), was cultured in Minimum Essential Medium (MEM) supplemented with 10% FBS, glutamine, and antibiotics. Cells were incubated at 37 °C in an atmosphere of 5% CO_2_.

To induce differentiation, the HOG cells were cultured in a differentiation medium (DM), which has been previously described [50]. All the experiments were performed with HOG cells previously cultured in DM for 24 h [50].

### 4.2. Viruses

Herpes simplex virus type 1 (HSV-1) was the wild-type F strain (the GenBank accession number for the DNA genome sequence is GU734771). GHSV-UL46, obtained from the American Type Culture Collection (ATCC), is a recombinant HSV-1 labeled by fusing the green fluorescent protein (GFP) to the tegument structural protein VP11/12, the product of the UL46 gene [51].

### 4.3. Isolation and Culture of Oligodendrocyte Precursor Cells (OPCs)

Extraction and culture of OPCs was performed as previously reported [33]. CD-1 mice (6–7 days old) were provided by the animal facility of the Centro de Biología Molecular Severo Ochoa-CBMSO (CSIC-UAM, Madrid, Spain). All animal procedures were performed in compliance with the European guidelines for Animal Research (European Communities Council Directives 2010/63/EU, 90/219/EEC, Regulation (EC) No.1946/2003) and were approved by the Ethical Review Board of Consejo Superior de Investigaciones Scientifics-CSIC and Comunidad de Madrid.

### 4.4. Generation of the LC3B Knockout HOG Cell Line

The Alt-R CRISPR-Cas9 crRNAs used were 5′-CGGCGACGACGCGAGGGUCCGUUUUAGAGCUAUGCU-3′ and 5′AGAUCCCUGCACCAUGCCGUGUUUUAGAGCUAUGCU-3′, which target exon 2 of the *MAP1LC3B* gene [52]. The crRNAs were mixed with Alt-R CRISPR-Cas9 tracrRNA and heated at 95 °C for 5 min. crRNA:tracrRNA duplexes were incubated with Alt-R S.p. HiFi Cas9 Nuclease V3 to assemble the ribonucleoprotein (RNP) complex. For RNP complex transfection, HOG cells were electroporated using the Alt-R Cas9 Electroporation Enhancer. Alt-R CRISPR-Cas9 system components were purchased from Integrated DNA Technologies (IDT) (Coralville, IA, USA). To grow single-cell clones, transfected cells were cultured in a 96-well plate at a density of 0.5 cells/well. Primer design, RNP transfection, and DNA sequencing were performed by the Transgenesis Core Facility at the Centro Nacional de Biotecnología (CNB, CSIC), Madrid, Spain. The absence of LC3B in knockout cells was verified by immunoblot analysis.

### 4.5. Antibodies

Primary antibodies included the rabbit polyclonal anti-LC3B (2220SS; Novus biologicals, Littleton, CO, USA), rabbit polyclonal anti-LC3B (ab51520; abcam, Cambridge, UK), rabbit monoclonal anti-LC3B (ab192890; abcam, Cambridge, UK), rabbit polyclonal anti-LC3A (AP1805a-EV; Abcepta, San Diego, CA, USA), rabbit polyclonal anti-LC3C (SAB4200822; Sigma, Saint Louis, MO, USA), mouse monoclonal anti-ICP8 major DNA-binding protein (ab20194; abcam, Cambridge, UK), rabbit monoclonal anti-lamin B1 (12586; Cell Signaling Technology, Danvers, MA, USA), mouse monoclonal anti-α-tubulin (T5168; Sigma, Saint Louis, MO, USA), mouse monoclonal anti-β-actin-peroxidase antibody (A3854; Sigma, Saint Louis, MO, USA), Alexa Fluor 647–phalloidin (A-22287; ThermoFisher, Waltham, MA, USA). Horseradish-peroxidase-conjugated secondary anti-IgG antibodies were purchased from Millipore (Billerica, MA, USA). Alexa Fluor secondary antibodies conjugated with fluorophores excitable at wavelengths of 594 or 488 were obtained from ThermoFisher Scientific (MA, USA).

### 4.6. Immunofluorescence Microscopy

Cell monolayers were grown on coverslips and differentiated for 24 h. After differentiation, cells were infected with HSV-1 at an m.o.i of 40. After 4–6 h, cells were fixed with 4% paraformaldehyde (PFA) in PBS for 15 min at room temperature (RT). Cells were then permeabilized with 0.2% Triton X-100, and non-specific antibody binding was blocked with 3% BSA and 10% human pooled serum (H4522; R&D Systems, Minneapolis, MN, USA) to block the HSV-1-induced IgG Fc receptors [53,54]. Cells were then incubated for 1 h at RT with the primary antibody diluted in PBS-BSA. The dilutions of the primary antibodies were as follows: anti-LC3B 2220SS (1:100), anti-LC3B ab51520 (1:200), anti-LC3B ab192890 (1:100), anti-LC3A (1:25), anti-LC3C (1:200), anti-ICP8 (1:200), and anti-phalloidin (1:100). After several washes, cells were incubated with Alexa Fluor secondary antibodies (1:500). Finally, DAPI was used to visualize the nuclei, and coverslips were mounted with Mowiol. Both DAPI and Mowiol were purchased from Calbiochem (San Diego, CA, USA).

### 4.7. Image Acquisition and Analysis

Images were acquired using a 63x oil immersion objective on an LSM900 confocal laser scanning microscope coupled with an upright Axio Imager 2 microscope (Zeiss, Oberkochen, Germany). Images were processed using ImageJ2 software. Traces were generated using the RGB profiler plugin. To quantify the intensity of fluorescence in the nucleus, the following computer code was used (see Supplementary Information). At least 150 cells from each condition were evaluated.

### 4.8. Immunoblot Analysis

HOG cells were differentiated for 24 h and then infected with HSV-1 at an m.o.i. of 40 for 6 h. Cells were lysed in Laemmli buffer (60 μM Tris-HCl, pH 6.8, 1% SDS, 6% glycerol). For the preparation of subcellular fractions, we used the Cell Fractionation Kit (90838S; Cell Signaling Technology, Danvers, MA, USA) according to the manufacturer’s instructions. Viscosity of the nuclear fraction samples was prevented by sonication with the UP50H Ultrasonic processor.

Cell lysates were reduced with 2-mercaptoethanol (5% *v*/*v*), subjected to SDS-PAGE in acrylamide gels, and transferred to Immobilon-P PVDF membranes (Millipore). The use of 15% acrylamide gels was required for the correct separation of LC3B forms I and II. After blocking with 5% nonfat dry milk and 0.05% Tween 20 in PBS, blots were incubated overnight at 4 °C with the corresponding antibody. The dilutions used for the antibodies were as follows: anti-LC3B 2220SS (1:500), anti-LC3B ab51520 (1:1000), anti-LC3B ab192890 (1:1000), anti-ICP8 (1:500), anti-lamin β1 (1:1000), anti-α-tubulin (1:500). After washing, the blots were incubated for 1 h at RT with a secondary antibody coupled to horseradish peroxidase. Anti-β-actin-peroxidase antibody (1:50,000) was directly incubated for 1 h at RT. Blots were revealed using an enhanced chemiluminescence Western blotting kit (ECL; Amersham, Little Chalfont, UK). Fiji-ImageJ software was used to quantify the abundance of autophagy markers and, subsequently, to calculate the ratio with respect to the loading control.

### 4.9. Transient Transfection of the GFP-LC3 Plasmid

The GFP-LC3 plasmid was generously provided by Natalia Reglero (CBMSO, CSIC, Spain). Cells were transfected with the plasmid using the X-tremeGENE 360 Transfection Reagent (Roche). The ratio of transfection reagent (µL) to DNA (mg) was 2:1. Cells were incubated with the reagent–DNA complex for 6 h. At 24 h after transfection, cells were fixed for immunofluorescence analysis.

### 4.10. Inhibition of HSV-1 Genome Replication

HOG cells were grown in 24-well plates and differentiated for 24 h. The cells were then infected with HSV-1 at an m.o.i. of 40 for 6h. Phosphonoacetic acid (PAA) (4408-78-0; Thermo Fisher Scientific, Waltham, MA, USA) was added to the medium at a concentration of 600 µg/mL at the time of virus adsorption and maintained throughout the course of infection.

### 4.11. Statistical Analysis

All statistical analyses were performed using GraphPad Prism (version 8.0.1; GraphPad Software, Inc., Boston, MA, USA). Data were subjected to Mann–Whitney U tests (non-parametric samples) to determine any significant differences between groups. A *p* value < 0.05 was considered statistically significant.

## 5. Conclusions

The use of polyclonal LC3B antibodies may result in non-specific staining in the nucleus of herpesvirus-infected cells. Caution should be exercised when interpreting LC3-immunofluorescence in viral infections. It is strongly recommended to validate these results using other complementary techniques, such as the analysis of LC3-II by immunoblotting.

## Data Availability

All the data generated or analyzed during this study are included in this published article.

## Supplementary Materials

The following supporting information can be downloaded at: https://www.mdpi.com/article/10.3390/ijms26146682/s1.
