# Supplementary material for: Polyclonal LC3B Antibodies Generate Non-Specific Staining in the Nucleus of Herpes Simplex Virus Type 1-Infected Cells: Caution in the Interpretation of LC3 Staining in the Immunofluorescence Analysis of Viral Infections
Source: Int J Mol Sci. 2025 Jul 11;26(14):6682. doi: 10.3390/ijms26146682 (PMC12294389; doi:10.3390/ijms26146682)
Supplement: Supplementary file 1 [file ijms-26-06682-s001.zip › CODIGO.pdf]

Supplementary information

\\ Computer code for quantifying the nuclear fluorescence intensity value

Cells were infected with HSV-1 for 6 hours, and then fixed with PFA 4%. Samples were stained anti-ICP8 (Alexa Fluor 488) and anti-LC3B (Alexa Fluor 555). Nucleus were stained with DAPI. This computer code serves to measure the intensity value of nuclear fluorescence for the channels 488 and 555, separately.

\\1.- Recursive function

```
function recursive(dirIn,dirOut) {

        lista = getFileList(dirIn); //lista = all images inside directory with images to process

        File.makeDirectory(dirOut); // check if results directory is created and if not create one.

        run("Set Measurements...", "area mean standard limit display redirect=None decimal=3");

        for (a=0; a<lista.length; a++){

// Loop to check the image format and to launch the treatment of the images one by one

// If there is any problem with the image format will be appear an error

                if(endsWith(lista[a],".vsi")){  //fichero formato vsi

                        run("Viewer", "open=["+dirIn+lista[a]+"]");

                        name = getTitle();

                        treatImage(name, dirOut); // Function for image treatment

                }

                else if (endsWith(lista[a],".tif") || endsWith(lista[a],".TIF") || endsWith(lista[a],".lsm")) { //fichero formato tif

                        open(dirIn+lista[a]);

                        name = getTitle();

                        treatImage(name, dirOut); // Function for image treatment

                }

                else if (endsWith(lista[a],".czi") || endsWith(lista[a],".lif") ){ //fichero formato czi o lif


                        run("Bio-Formats Windowless Importer", "open=["+dirIn+lista[a]+"]");

                        name = getTitle();

                        treatImage(name, dirOut); // Function for image treatment

                }

                else{

                        print("Esto no es una imagen.");

                }

        }
```

```
        if (isOpen("Results")){

                selectWindow("Results");

                saveAs("Results", dirOut + "Results.xls");

        run("Close");

    }

    if (isOpen("Summary")){

                selectWindow("Summary");

        run("Close");

    }

        if (isOpen("ROI Manager")) {

        selectWindow("ROI Manager");

        run("Close");

    }

    if (isOpen("Log")) {

        selectWindow("Log");

        run("Close");

    }

        run("Close All");

//Export the results as an Excel (xls format) and close all the windows that have been
opened

        Dialog.create("Final");

        Dialog.addMessage("Usted por hoy ha terminado y puede irse a dormir la
siesta");// MENSAJE DESPEDIDA

        Dialog.show();

}

// Final message to end up the program
```

\\2.- Image treatment

```
function treatImage(nameOri, dirOut) {

        getDimensions(width, height, channels, slices, frames);

        if(slices >1){

                run("Duplicate...", "title=img1 duplicate slices=2");

        }

        else {

                rename("img1");

        }

        run("Split Channels");

        selectWindow("C1-img1");

// Get the image dimensions and split the different channels, which will be renamed

        run("Duplicate...", "title=img2 duplicate");

        selectWindow("C1-img1");
```

```
        setAutoThreshold("Otsu dark");

        //run("Threshold...");

        setOption("BlackBackground", true);

// Set the threshold "Otsu dark" to select the nucleus

        run("Convert to Mask");

        run("Dilate");

        run("Fill Holes");

        run("Median...", "radius=2");

// Nucleus were stained with DAPI, however, regions with less chromatin were not stained effectively. To measure the fluorescence in the entire nucleus, the program fills the unstained holes within the nucleus.

run("Analyze Particles...", "size=12-Infinity exclude add");

// Selection of the pixel size of rendered image (nm). Particles outside the range specified in the Size field are ignored.

        selectWindow("C2-img1");

        rename(nameOri+"_488_");


        if(channels ==3){

                selectWindow("C3-img1");

                rename(nameOri+"_555_");

        }

        else if (channels == 4){

                selectWindow("C4-img1");

                rename(nameOri+"_555_");

        }

        else{

                print("ERROR!: Faltan canales.");

                break;

        }

// Selection and renamed of the channels 488 and 555.

// Images can have 3 (C1-DAPI, C2-488, C3-555) or 4 channels (C1-DAPI, C2-488, C3-bright field, C4-555). If there is not the case of the image, the program alerts the error.


        n = roiManager("count");


        for(i=0; i<n; i++){

                selectWindow(nameOri+"_488_");

                roiManager("select", i);

                //run("Restore Selection");

                run("Measure");

        }

        for(j=0; j<n; j++){

                selectWindow(nameOri+"_555_");
```

```
                roiManager("select", j);
                //run("Restore Selection");
                run("Measure");
        }
// Quantification of the intensity fluorescence value of the channels 488 and 555, separately, by the roiManager.

        selectWindow(nameOri+"_555_");
        rename("555");
        selectWindow(nameOri+"_488_");
        rename("488");
        run("Merge Channels...", "c1=555 c2=488 c3=img2 create ignore");
        roiManager("Show All with labels");
        run("Flatten");
        saveAs("Tiff", dirOut +"Result_"+nameOri);
// The channels 488 and 555 are merged. The number associated to each nucleus is added to the image. The labels are fixed to the image with the command “flatten”. The image is saved as a tiff in the directory of the results.
        run("Close All");
        run("Select None");
        selectWindow("ROI Manager");
        run("Close");
// The windows of the ROI Manager are closed.

}
```

//3.- Program launch

```
setOption("ExpandableArrays", true);
abrir=getDirectory("Elige el directorio donde están las imágenes originales");
guardar=getDirectory("Elige el directorio donde deseas almacenar los resultados");
almacen = guardar + "Resultado"+ File.separator;
// abrir = directory with images to process
// guardar = directory to save results
// almacen = path to créate directory inside guardar with Name Results
recursive(abrir,almacen); //Call recursive Function
```
