# Supplementary material for: Polyclonal LC3B Antibodies Generate Non-Specific Staining in the Nucleus of Herpes Simplex Virus Type 1-Infected Cells: Caution in the Interpretation of LC3 Staining in the Immunofluorescence Analysis of Viral Infections
Source: Int J Mol Sci. 2025 Jul 11;26(14):6682. doi: 10.3390/ijms26146682 (PMC12294389; doi:10.3390/ijms26146682)
Supplement: Supplementary file 1 [file ijms-26-06682-s001.zip › SUPLEMENTARIO.pdf]

Supplementary material

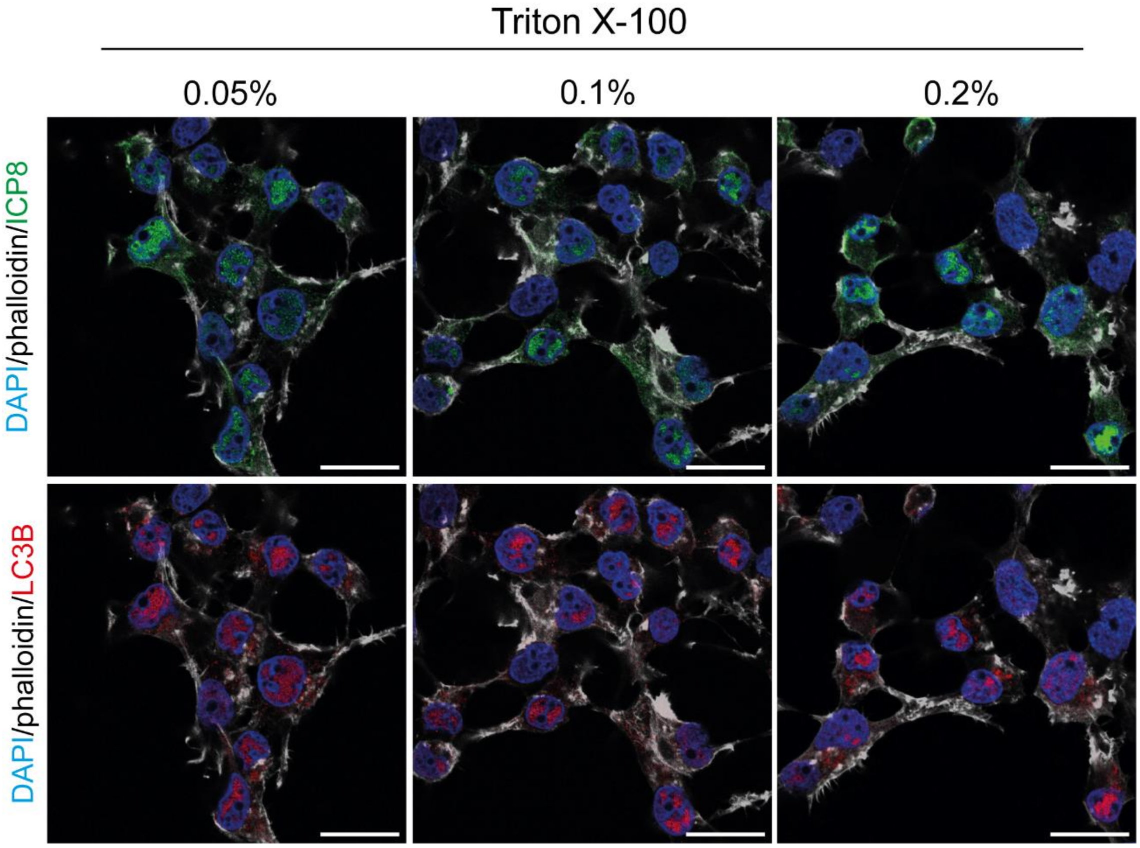

Figure S1. Detection of LC3B staining in HSV-1 infected cells using different percentages of Triton X-100 for permeabilization. HOG cells were infected with HSV-1 and fixed at 6 hpi. The samples were permeabilized for 5 min with percentages of Triton X-100 ranging from 0.05% to 0.2 % and then probed with anti-ICP8 and anti-LC3B (2220SS). Nuclei were labeled with DAPI and the contour of the cells was visualized with Fluor 647-phalloidin. Scale bar, 20 μm.

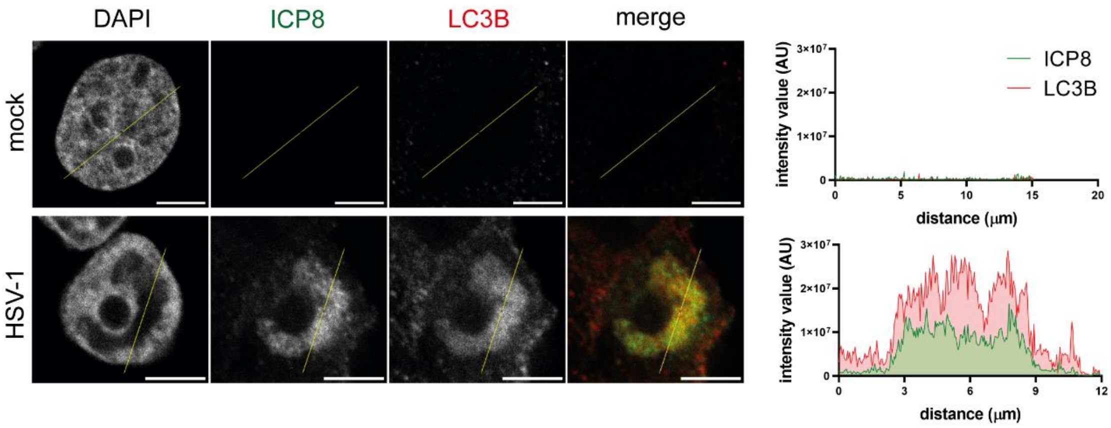

Figure S2. LC3B staining in the nucleus of HSV-1 infected ATG5 KO cells. ATG5 KO HOG cells were infected with HSV-1 and fixed at 6 hpi. The samples were probed with anti-ICP8 and anti-LC3B (2220SS). Nuclei were labeled with DAPI. The graphs show the intensity value of ICP8 and LC3B fluorescence across the yellow line of the images. AU, arbitrary unit. Scale bar, 5 μm.

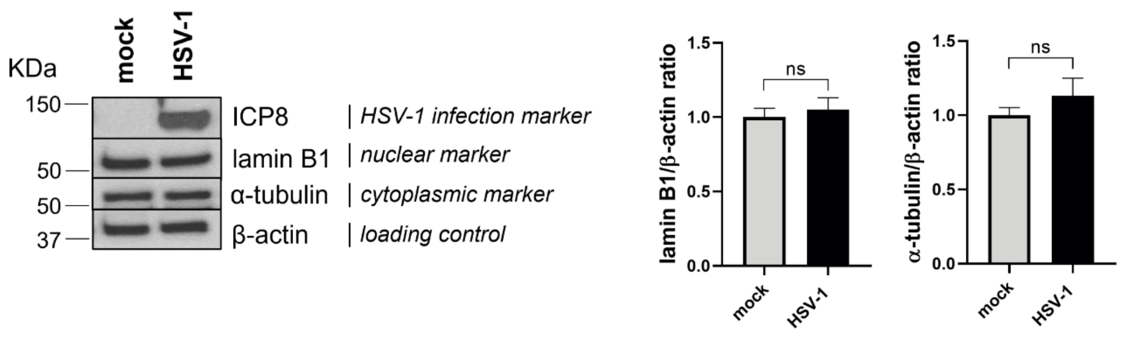

Figure S3. Lamin B1 and α-tubulin can be used as loading controls in immunoblot analysis of HSV-1 infected cells. HOG cells were infected with HSV-1 and the cell lysates were collected at 6 hpi. The protein levels of lamin B1 and α-tubulin were measured by immunoblot analysis. β-actin was used as loading control. The data were normalized to the value of mock-infected cells (set to 1) and reported as the mean ± SEMs. The comparison between the data of infected cells and the corresponding mock controls (n=4) was not statistically significant (ns) according to the two-tailed Mann-Whitney test. As lamin B1 and α-tubulin remain unchanged during HSV-1 infection, they can be used as loading controls in immunoblotting.

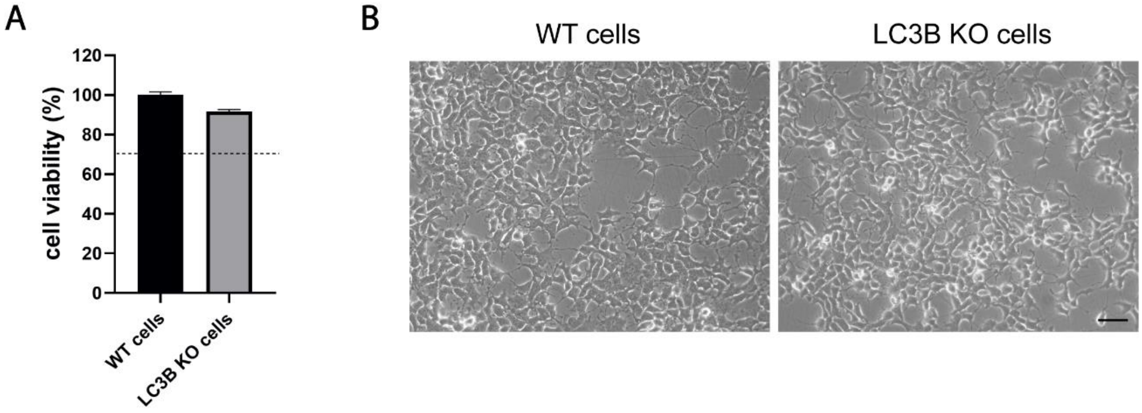

Figure S4. Validation of cell viability and morphology of LC3B knockout (KO) HOG cells. (a) Wild-type (WT) and KO LC3B HOG cells were culture in a 96-well plate for 24h and the cell viability was determined by an MTT assay (Promega, Cell Titer 96 Non-Radiactive Cell Proliferation Assay). The readouts obtained from the MTT assay were normalized to the value of WT cells (setting to 100%). The cellular viability of KO cells remained above 90 %. **(b)** Optical images of WT and LC3B KO cells after 24h in DM. The Olympus CKX41 microscope with a 20X objective was used to capture the images. No changes were observed in HOG cell morphology in the absence of LC3B. Scale bar, 50 μm.

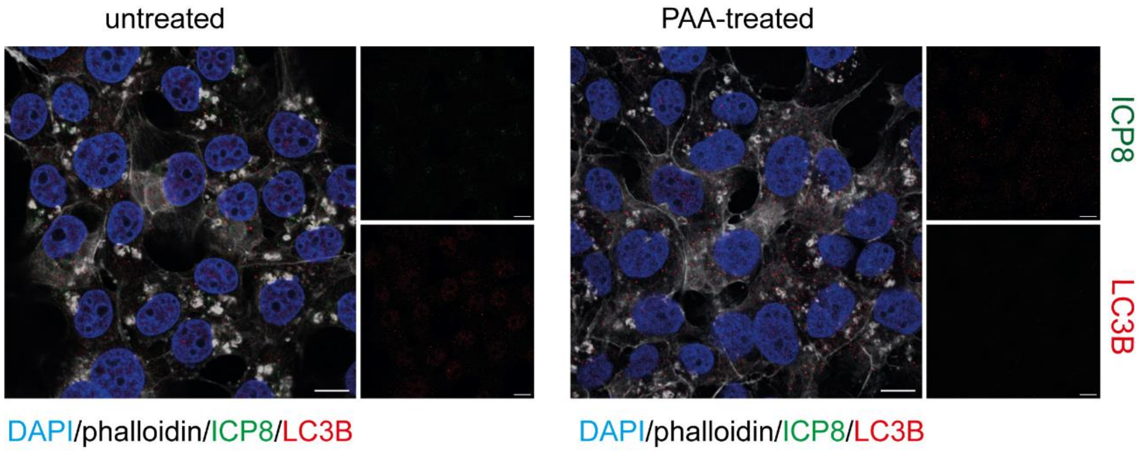

Figure S5. LC3B staining is not detected in PAA-treated HOG cells. HOG cells treated with 600 μg/ml PAA for 6h. Untreated cells were used as control. All samples were probed with anti-ICP8 and anti-LC3B (2220SS). Nuclei were labeled with DAPI and the contour of the cells was visualized with Fluor 647-phalloidin. Scale bar, 10 μm.

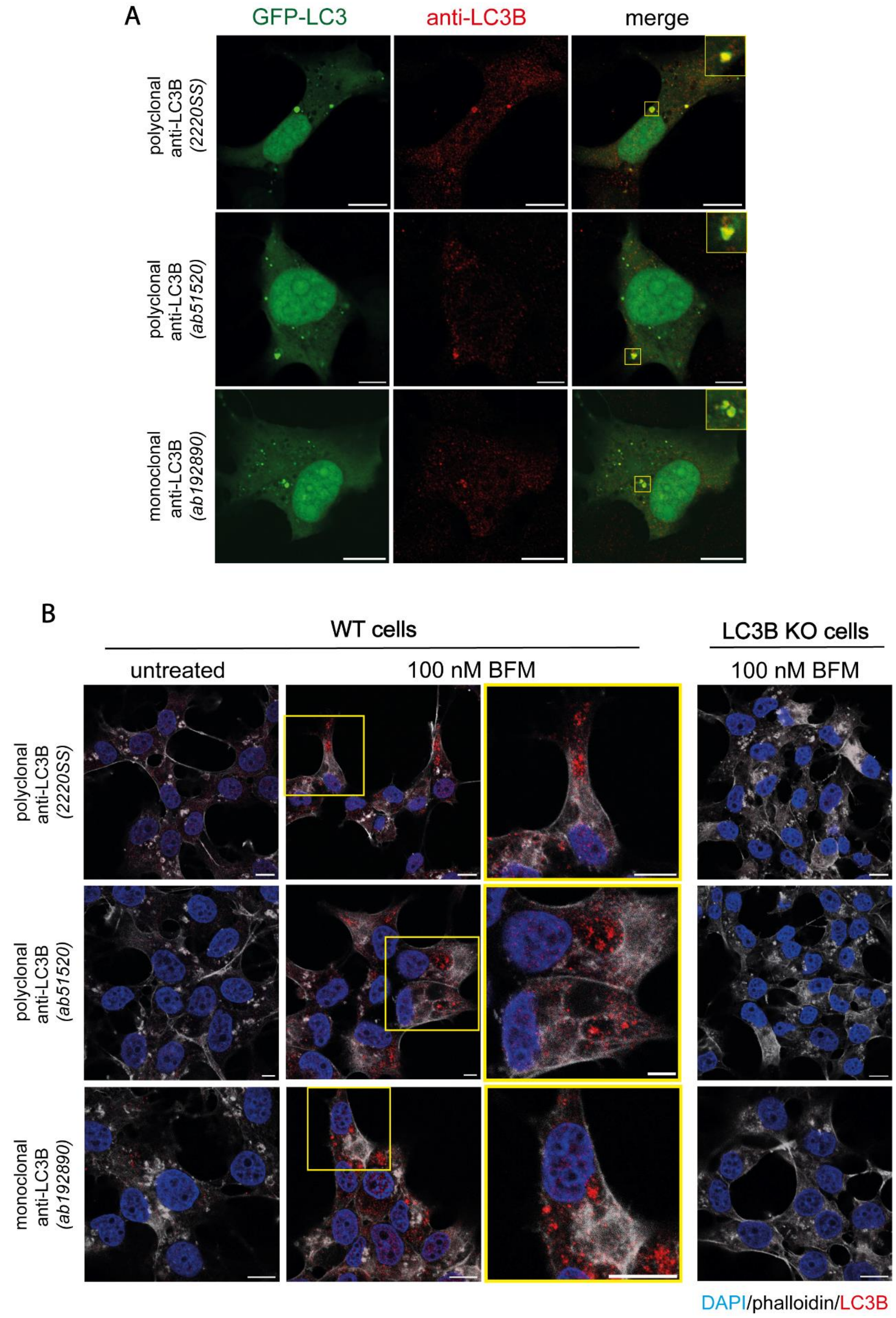

Figure S6. LC3B antibodies are suitable for the LC3-immunofluorescence analysis in uninfected cells. **(a)** HOG cells were transiently transfected with a GFP-LC3 plasmid. After transfection, cells were fixed and stained with the different LC3B antibodies. **(b)** The cells were treated with 100 nM BFM (B1793, Sigma) for 24h and, after fixation, they were probed with LC3B antibodies. Alexa Fluor 647-phalloidin was used to visualize the contour of the cells and nuclei were labeled with DAPI. Scale bar, 10 μm.
