# Supplementary figures and images for: Polyclonal LC3B Antibodies Generate Non-Specific Staining in the Nucleus of Herpes Simplex Virus Type 1-Infected Cells: Caution in the Interpretation of LC3 Staining in the Immunofluorescence Analysis of Viral Infections
Source: Int J Mol Sci. 2025 Jul 11;26(14):6682. doi: 10.3390/ijms26146682 (PMC12294389; doi:10.3390/ijms26146682)

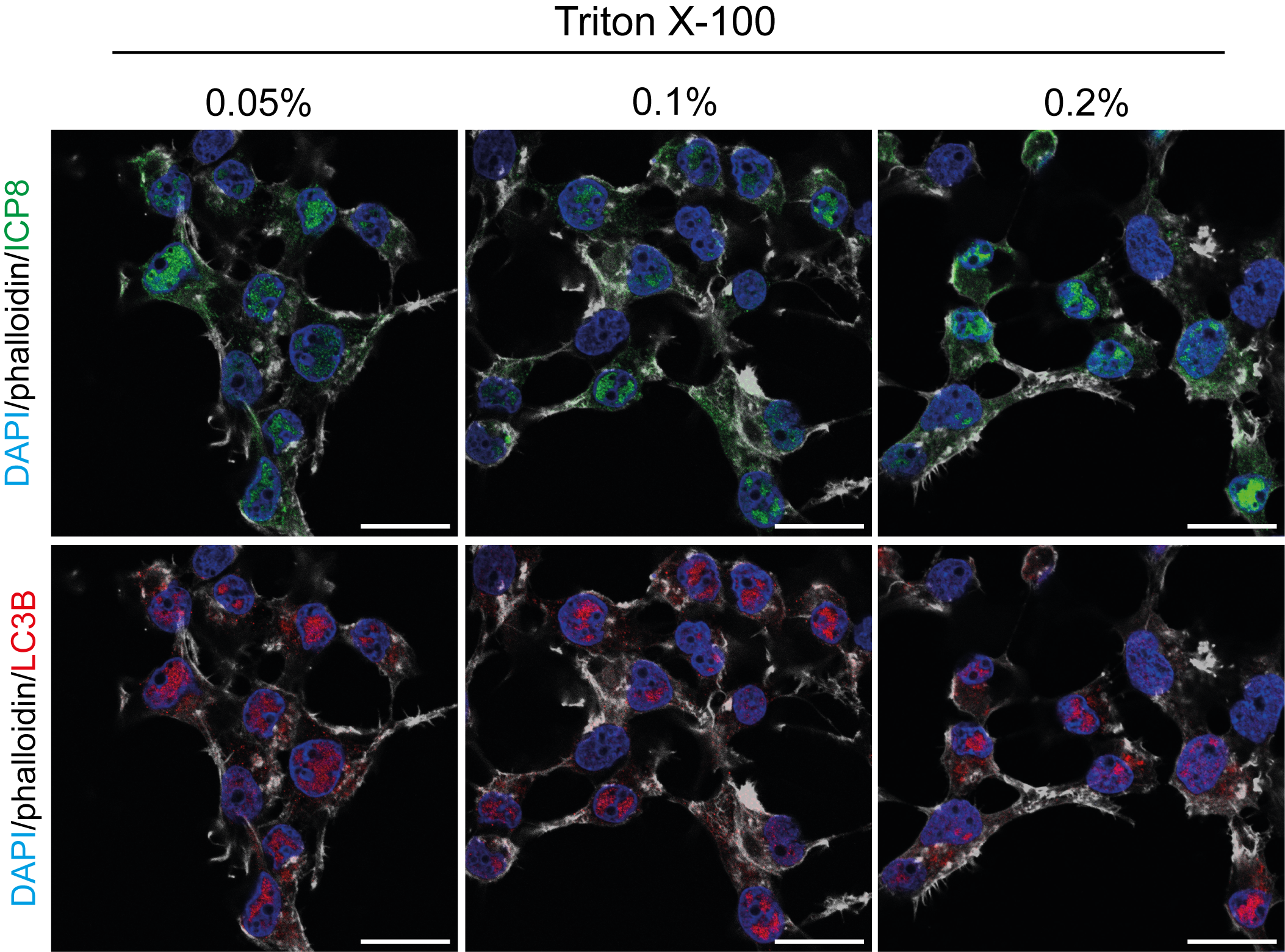

Supplement: Supplementary file 1 [file ijms-26-06682-s001.zip › FIG S1.tif]

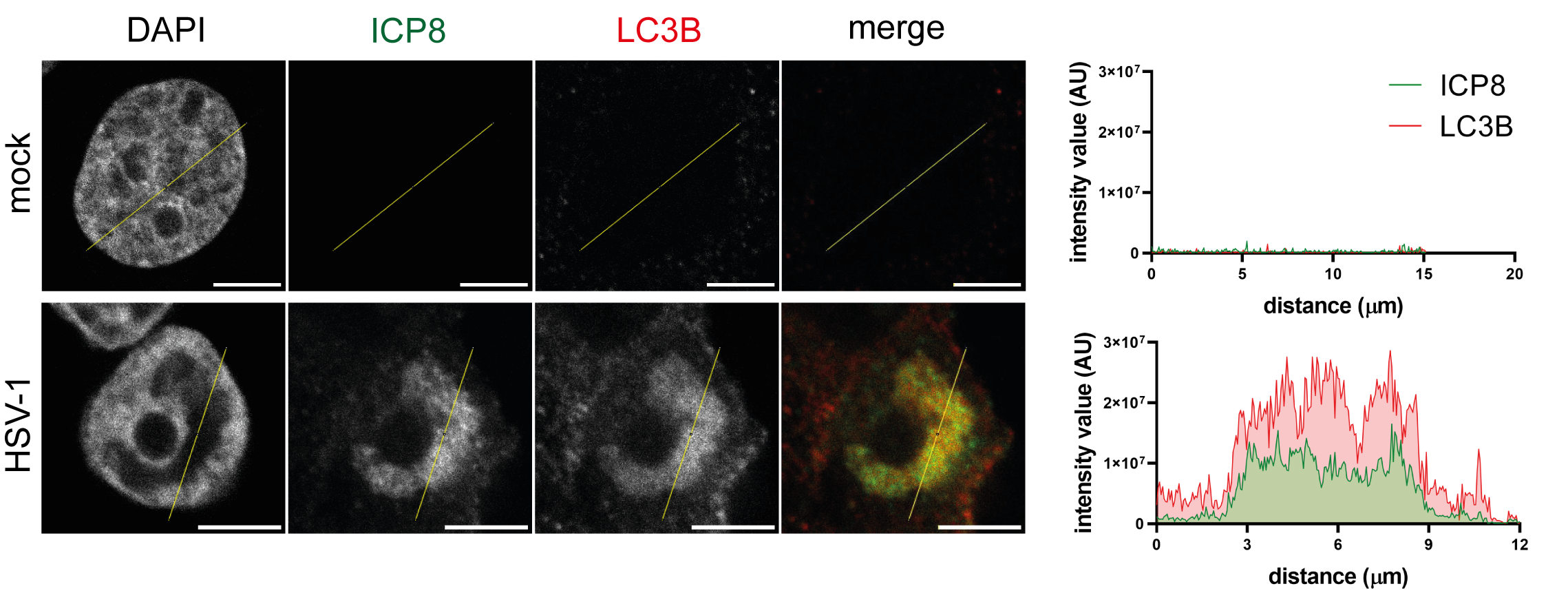

Supplement: Supplementary file 1 [file ijms-26-06682-s001.zip › FIG S2.tif]

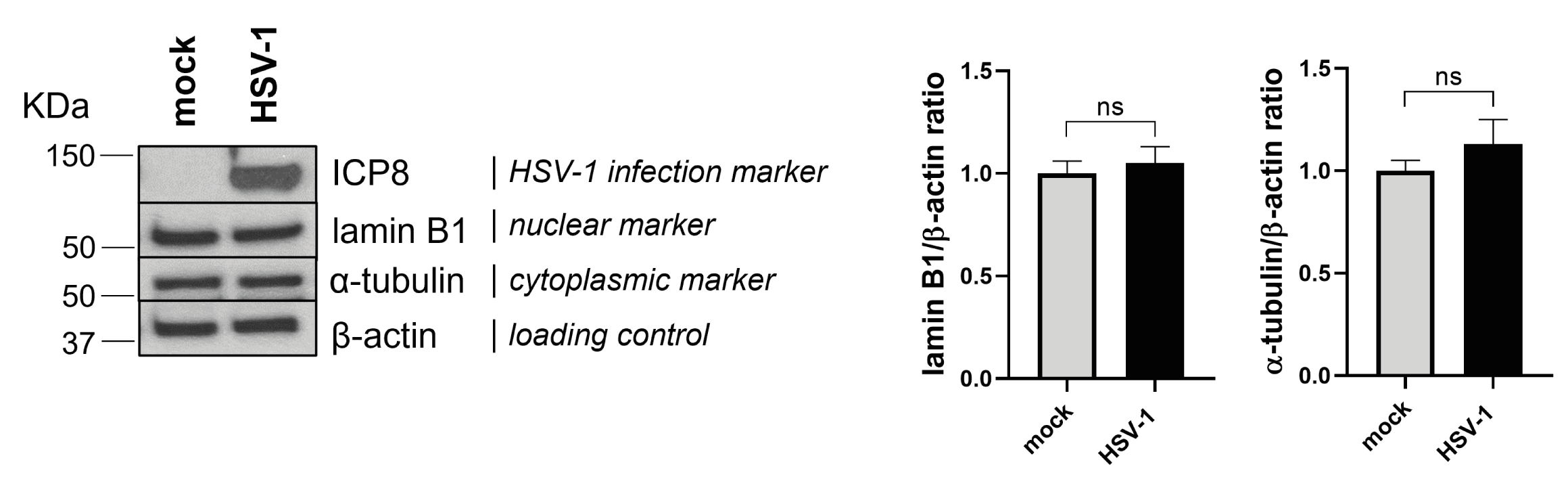

Supplement: Supplementary file 1 [file ijms-26-06682-s001.zip › FIG S3.tif]

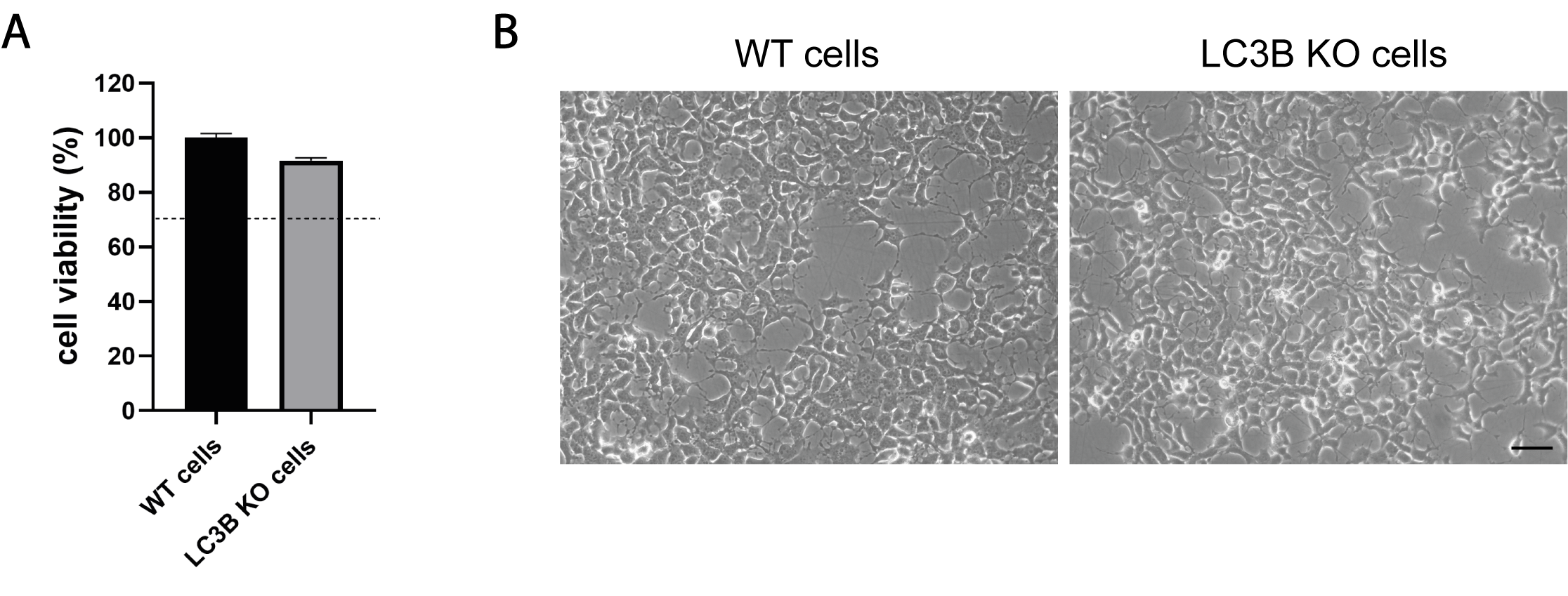

Supplement: Supplementary file 1 [file ijms-26-06682-s001.zip › FIG S4.tif]

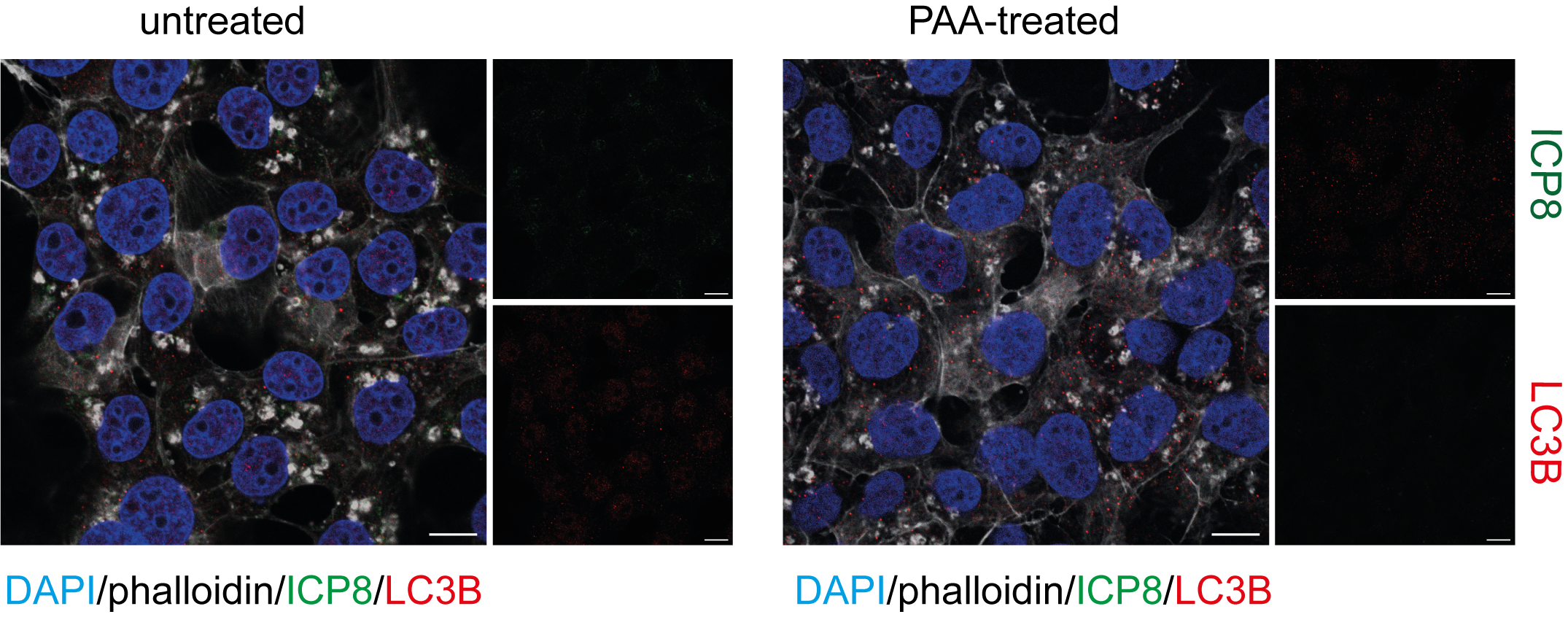

Supplement: Supplementary file 1 [file ijms-26-06682-s001.zip › FIG S5.tif]

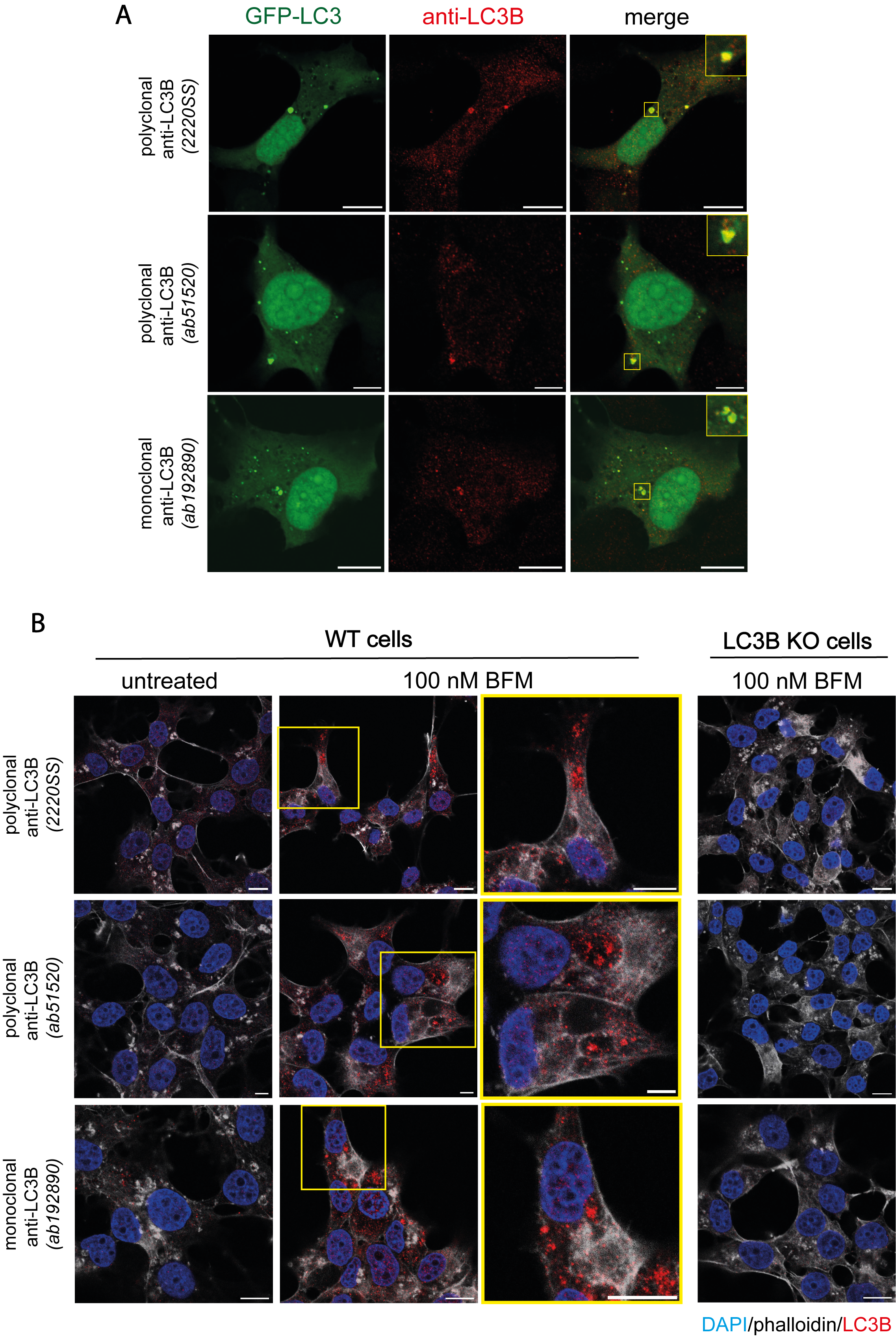

Supplement: Supplementary file 1 [file ijms-26-06682-s001.zip › FIG S6.tif]
